# Cross-cultural metathemes of Chinese and Japanese university students' perspective on parental care

**DOI:** 10.3389/fpubh.2023.1216831

**Published:** 2023-09-04

**Authors:** Xuxin Peng, Hisae Nakatani, Huifang Chen, Yuriko Inoue, Fang Song, Mikako Yoshihara, Ruxin Lei

**Affiliations:** ^1^Department of Community and Public Health Nursing, Graduate School of Biomedical and Health Sciences, Hiroshima University, Hiroshima, Japan; ^2^School of Nursing, Guangzhou Medical University, Guangzhou, Guangdong, China

**Keywords:** parental care, health policy, cross-cultural study, China, Japan

## Abstract

**Introduction:**

Due to declining birthrates and aging populations, parental care is going to place a greater burden on younger generations in the future, especially in East Asia where it is more common for children to provide care regardless of whether there is a national long-term care insurance program. Therefore, it has become important to understand the younger generation's views on parental care.

**Methods:**

An explorative, metathematic qualitative study design was used. Data collection relied on semi-structured interviews, of which 19 Chinese and 19 Japanese university students were conducted from December 2021 to July 2022 using a snowball sampling method. Metatheme analysis was then used to identify broad cross-cultural metathemes and inter-relationships on parental care.

**Results:**

Three parental care metathemes were identified for the perspectives of parental care: distrust of leaving parental care to others, responsibility to care for their parents, and importance of parent-child interactions about parental care.

**Conclusion:**

To improve social support for care, both countries must improve long-term care service delivery and healthcare systems and ensure that there is a trusting relationship between healthcare professionals and the public. Governments should also ensure that adult children receive assistance to balance their work, life, and parental care responsibilities. The findings provide several practical suggestions for improving healthcare systems in China and Japan through the younger generations' views.

## 1. Introduction

Japan has an aging population and a declining birthrate. Currently, Japan has the highest global percentage of people over 65 years old (28.4%), which is expected to rise to 38.3% by 2055 (1). Japan's total fertility rate has also fallen from 2.14 in 1970 to only 1.42 in 2018 and has continued to decline (2). Meanwhile, China has a rapidly aging society and a declining birthrate, the latter of which is falling faster than in Japan. In 2022, over 13.7% of China's population was over 65, which is expected to rise to 30.1% by 2050 (3). China's 1979 one-child policy has led to the 4-2-1 family, that is, single-child couples must care for four older adults and their child (4). As China's birth rate is now at a historic low (5), the government has introduced a three-child policy to encourage families to have more children; however, many young parents find that the cost of having children is too high and tend to only have one or two children. Consequently, parental care for this generation will be a serious social problem in the coming decades (6).

A strong sense of filial piety continues to exist in Asian countries (7). In Japan, a highly developed country, traditional norms of filial piety coexist with new independence norms. Because of Japan's significant demographic changes, that is, a rapidly aging population and declining birth rates, many children are expected to have difficulty caring for their parents, meaning that the public service need will continue to increase (8). In China, traditional Chinese norms of filial piety mean that to ensure continued happiness, children must respect their parents, live with them, and care for them, all of which involve sacrifice (9). However, due to rapid socioeconomic development, modernization, and globalization, Chinese perceptions and values regarding filial piety are changing and more social resources and cultural support for older adults are now being provided (10, 11).

Japan's long-term care insurance system, which was established in 2000, was implemented under the slogan “from care by the family to care by society,” (12); and China's “Guidelines for promoting the development of national undertakings for the older adults and improving the long-term care service system during the 14th Five-Year Plan period (2021–2025),” which was submitted by the State Council of China in 2021, advocates both family and societal support for the older adults by strengthening community care service capacity and establishing a long-term care insurance system similar to the system in Japan (13).

The centering of the parent-child relationship in Japanese society means that children have obligations and responsibilities to their parents, which means the Japanese need to take care of their parents (14). Also, in China, because of the previous lack of a functioning long-term care system, young Chinese have been the main caregivers for older adults, and, even today, 90% of older adults are still dependent on familial care (15).

Therefore, this study examined the views of Japanese and Chinese university students to identify the commonalities and differences in family care and social culture values, the results from which could guide future healthcare system needs. These days, university students are less influenced by parental and secular influences and more by their peers. Therefore, it was expected that they may have different perspectives regarding parental care to their parents and would be more likely to be more hesitant in following traditional parental care norms (16).

Previous studies have found that parental care perspectives have changed rapidly over the past few decades, which has raised concerns about the future of parental care by the generation born under the one-child policy (17). For example, Bifarin et al. found that the single-child Chinese student perspective of parental care in the UK was related to family obligations and filial piety expectations, which emphasized the importance of cross-border and cross-cultural exchanges (18). Although filial piety remains rooted in the Chinese psyche, further studies are needed to compare China with other Confucian-influenced Asian countries undergoing social, demographic, and economic changes (19). On the other hand, despite demographic changes, socio-economic development, and Long-Term Care reforms in Japan influencing traditional caregiving values, parental care is an important but unresolved issue (20). Tanaka et al. found that caregiving sons experience psychological distress, leading to high rates of abuse of their parents (21). Studies highlight the importance of adult children, particularly the oldest son, as caregivers for their parents in Japan (22). Conversely, the declining trend of bequests for altruism in Japan resulted in the increasing use of care services (23), and parental caregiving behavior of Japanese adult children is also heavily influenced by both the strength of altruism and the strategic bequest motive (24). However, there are relatively few historical studies that focus on the comparison of the younger generation's perspectives between different countries. Therefore, this study integrated the parental care perspectives of adult Japanese and Chinese university students to reveal the steps necessary to allow similar societies to examine their care practices.

Declining birthrates, aging populations, and conflicts between traditional norms and public parental care policies have become major social issues in China and Japan. Therefore, this study sought to identify the parental care perspectives of Chinese and Japanese university students to guide the development of possible healthcare programs that could provide better future parental care.

## 2. Methods

### 2.1. Design and participants

This exploratory study adopted a metatheme approach using semi-structured interviews. Metatheme is a qualitative method for cross-cultural research, which provide an analytically significant pattern to describe those patterns in rich, contextually appropriate ways within cross-cultural data sets (25).

Participants were recruited from University A in Japan and university B in China. Inclusion criteria comprised enrolled Japanese and Chinese adult university students. The potential subjects were approached from the referral of the teachers in University A and B, who only introduced the purpose of the study and were blind to the study procedure for diminishing possible conflicts of interest. A snowball sampling method was used, whereby eligible participants were asked to link the researchers to other potential participants. Students who expressed an interest to participate received an information sheet. Students had at least 2 weeks to consider the participant after receiving the information sheet and being informed that their participation was entirely voluntary.

Twenty-one Japanese potential participants and 19 Chinese potential participants expressed interest. Two Japanese students declined for time constraints. As a result, 19 adult Japanese university students at University A in Japan and 19 adult Chinese university students from University B in China were recruited, which exceeded the minimum number needed for metathematic qualitative research saturation (26). The number of participants in each school year was also balanced to ensure accurate data representation. The participants were divided into four Japanese focus groups and two Chinese focus groups according to the participant's willingness and schedules.

### 2.2. Data collection

Data were collected from December 2021 to July 2022. Before the interviews, the study participants were informed that their participation was entirely voluntary again. Two interviewers then conducted 80-min focus group interviews (FGI) that followed the consolidated criteria for reporting qualitative research (COREQ) guidelines (27). The interviews were digitally recorded. During the FGIs, two researchers participated in Japanese focus groups and three researchers participated in Chinese focus groups as observers. The observers record the data and non-verbal communication such as facial expressions and raising hands to maintain a heightened level of perspective (28). To identify and compare their perspectives, it isn't allowed researchers' beliefs and assumptions to shape the process of data collection (29). Interviewers and observers were fully trained in interview methods before the FGIs (30). During the FGIs, interviewers and observers paid attention to participants' wellbeing, if anyone shows or expressed signs of discomfort, the FGI would be paused immediately.

The FGIs, which were conducted at the participants' respective universities, focused on several main questions, namely, how do you feel about taking care of your parents? What is important to you when caring for your parents? Finally, what are your perceptions and opinions regarding the use of care services to care for your parents vs. taking care of them yourself?

### 2.3. Analysis

All FGIs were recorded with the written informed consent of the participants, from which transcriptions were made. Metatheme analysis was then used to identify the broad cross-cultural metathemes and inter-relationships (25).

The data analysis was conducted as follows. First, each transcript was repeatedly read to ensure familiarization and gain an overall sense of the data. The materials were then categorized and separated into fundamental units for each country that were relevant to the study goals. After the thematic analysis was completed, a list of themes for each country was developed and the metathemes were identified. To ensure that the culturally situated meanings of the site-specific themes were not lost, all themes were translated into Japanese by researchers who could speak both Chinese and Japanese. After further comparison of the similarities and differences, sub-metathemes were then developed for the related or comparable codes, the classifications for which were developed by comparing and grouping the subcategories. After that, the metathemes were compared and distilled into a smaller set of metathemes. After multiple review rounds, further convergent metathemes and sub-metathemes were developed.

To ensure analytical rigor, Lincoln and Guba's four-dimensional criteria were used: (1) credibility, that is, checking the study participants who cooperated; (2) transferability, that is, describing in detail the phenomena obtained from the analysis; (3) dependability, that is, specifying the entire data collection and results acquisition process in a report; and, (4) confirmability, that is, conducting continuing discussions between the co-researchers and the research team to decide on the final themes with the assistance of two public health and qualitative research experts (31).

### 2.4. Ethics

Approval from the Ethics Committee of the epidemiology research department at our university was obtained. All procedures were conducted in line with the Helsinki Declaration. An information sheet explaining the study's goals, procedures, and ethical considerations was given to each participant, which also explained that they could discontinue or withdraw from the study at any time, that the interview would be recorded and their anonymity would be preserved, and that the study may be published in journals or presented at conferences. Participant agreement was obtained by signature. On the day of the FGIs, the researchers repeated this information before the interview and received verbal agreement again from the participants.

## 3. Results

### 3.1. Participant characteristics

Interview data were obtained from 19 Japanese university students and 19 Chinese university students. The participants were divided into four Japanese groups and two Chinese groups. The mean age of the Japanese students was 20.94 (SD = 3.05), and the mean age of the Chinese students was 20.84 (SD = 1.68). Table 1 gives the basic participant attributes.

**Table 1:** Participant characteristics.

**JPN No**.	**Gender**	**Grade**	**Age**	**Birth order**	**CN No**.	**Gender**	**Grade**	**Age**	**Birth order**
J-1	Female	4	23	First-born child	C-1	Female	1	19	Only child
J-2	Male	4	22	First-born child	C-2	Female	1	18	First-born child
J-3	Female	3	21	First-born child	C-3	Female	2	20	Only child
J-4	Female	4	22	Not first-born child	C-4	Female	2	20	Only child
J-5	Male	4	23	Not first-born child	C-5	Female	4	22	Only child
J-6	Female	4	21	First-born child	C-6	Male	4	23	Only child
J-7	Female	3	20	First-born child	C-7	Male	3	21	Only child
J-8	Female	3	20	First-born child	C-8	Female	2	20	First-born child
J-9	Female	3	21	Not first-born child	C-9	Female	3	20	Only child
J-10	Female	3	21	Not first-born child	C-10	Female	3	21	Not first-born child
J-11	Female	3	20	First-born child	C-11	Male	3	23	First-born child
J-12	Female	2	19	Not first-born child	C-12	Female	2	20	Only child
J-13	Female	1	19	First-born child	C-13	Female	1	19	Not first-born child
J-14	Female	2	19	First-born child	C-14	Female	4	22	First-born child
J-15	Male	1	19	Only child	C-15	Male	4	24	Only child
J-16	Male	1	19	Not first-born child	C-16	Male	3	22	Only child
J-17	Male	1	18	First-born child	C-17	Male	2	20	Not first-born child
J-18	Male	1	32	First-born child	C-18	Female	4	23	Not first-born child
J-19	Male	1	19	Only child	C-19	Male	1	19	First-born child

JPN, Japan; CN, China.

Japan: Group 1: J-1 J-6: Group 2: J-7 J-9: Group 3: J-10 J-14: Group 4: J-15 J-19.

China: Group 1:C-1 C-9;Group 2:C-10 C-19.

### 3.2. Parental care

Three metathemes were identified for parental care: distrust of leaving parental care to others, responsibility to care for parents, and the importance of parent-child interactions about parental care.

#### 3.2.1. Distrust of leaving parental care to others

This metatheme comprised four sub-metathemes (Table 2).

**Table 2:** Metatheme 1: distrust of leaving parental care to others.

**Sub-metathemes**	**Themes**	
	**China**	**Japan**
Poor impression of nursing homes	I would not leave my parents in a nursing home because I have many bad impressions of nursing homes	I have seen many incidents of abuse and violence in the news, and I'm distrustful of whether my parents could stay in a nursing home
	I am not comfortable with the atmosphere in a nursing home, so I would not leave my parents in their charge	
Doubts about the competency and professionalism of caregivers	If you leave a patient with a caregiver, I have doubts about their professionalism	I do not know if the caregivers' education level is improving, so I am not sure if they would be able to properly care for my parents
	I do not trust the professionalism of caregivers	
Distrust about the attitudes of caregivers	I do not trust the caregivers because they are not my family	I do not know if the caregiver is motivated, and I distrust them to care for my parents
	Caregivers are not wholehearted	
	When a caregiver cares for a parent, it is not full-service	
Fewer care workers are available on caring for their parents	I can rely on others to care for my parents only if the number of caregivers increases	The number of caregivers is less than the number of people who need care; therefore, I am concerned about whether they could properly care for my parents
	Not enough caregivers to properly care for my parents	Feel unable to properly care for their parents due to a lack of manpower and too much work to do

Because both the Chinese and Japanese participants had poor impressions of nursing homes, they had a distrust of leaving parental care to nursing homes.

The atmosphere in the nursing home is not particularly good, and I don't think my parents would be properly cared for if they lived there. If children are forced to leave their parents in a nursing home, the parents will feel as if they have been abandoned by their children. (C-2)
I have seen the daily news about nursing homes, about the abuse and neglect by caregivers. After I heard about such a tough situation, I could hardly believe that my mother would be properly cared for in a facility. (J-9)

Participants from both China and Japan had doubts about the competency and professionalism of nursing home caregivers to take care of their parents.

I doubt the expertise of the caregiver. I want to care for my parents by myself as much as possible, as being cared for by a caregiver who lacks expertise may cause irreversible aftereffects. And they cannot grasp the psychological needs of my parents. (C-4)
I don't know if the education level of the caregivers is getting better, but as long as it's left to those people, I'd be somehow worried......So, I think I'm a little distrustful that they will be able to properly care for my parents. (J-10)

Both the Chinese and Japanese participants distrusted the attitudes of the caregivers. The Chinese participants felt that the caregivers were not part of the family, and thought that caregivers might have negative thoughts, which would lead to distrust. The Japanese participants were doubtful and distrustful because they wondered whether the caregivers were motivated to be caregivers as this work was not an elite job.

As caregivers are not family members, I do not feel secure that they could provide fully comprehensive care for my parents... I want to care for my parents by myself because if I leave them in a nursing home, I am afraid they might be abused, and that the caregivers would not be as caring as I would like them to be. (C-7)
The caregivers entrusted to do the job should be highly motivated, but the fact is, those people have various problems (pay, stress) …For situations like this, I am not sure whether they would be motivated to care for my parents. So, I guess I'm a little distrustful at this stage. (J-10)

the Chinese or Japanese participants trusted the caregivers because there were few caregivers and they had a lot of work to do. As there were not enough caregivers for all the people who needed care, they felt anxious about the caregivers' abilities to care for their parents.

Because a large number of older adults need to be cared for by a single caregiver, if my parents were left in the care of a facility, no one could ensure that they would be properly cared for. If the number of caregivers was increased, it would be feasible for me to leave my parents in a nursing home. (C-14)
The number of caregivers is not enough for the people who will need care in the future. I am worried that my parents would not be cared for properly in this situation. (J-6)

In addition, this metatheme reflected the participants' distrust of leaving parental care to others. Even though caregivers and nursing homes exist, the Chinese and Japanese participants expressed their distrust of them, highlighting that there were several barriers to caregivers and nursing homes gaining their trust.

#### 3.2.2. Responsibility to care for their parents

This metatheme comprised four sub-metathemes (Table 3).

**Table 3:** Metatheme 2: responsibility to care for parents as their child.

**Sub-metathemes**	**Themes**	
	**China**	**Japan**
Responsibility to care for their parents	Following the principles of filial piety, all parental care is the responsibility of the child	When I became an older brother, I was taught that I had to be a good older brother
	The “firstborn concept” is an old belief and needs to be discarded	Gender equality in social participation is widespread, but the impression remains that care should be done by women
	All children should be responsible for parental care for fairness	I still have the impression that my sister would do all the work for me
Repayment for bringing up child	I have a responsibility and an obligation to care for my parents to repay their upbringing	I will take care of my parents because they raised me so far
	Responsibility to care for my parents recoups their cost of raising me	
Influence from surroundings	I want to care of my parents by myself because it is my filial duty	I hate to start bad rumors about me if I sent my parents to a nursing home
	Influenced by idioms like “growing up children, old people's security,” I think it is my responsibility to care for my parents	If my parents enter a group home, people might think I am not fulfilling my role as a child, so I will take care of my parents
	I want to care for my parents myself because it is important for me to keep my filial piety.	
Balance between work and care	Family is more important than work	If it's hard to take time off from my job when I need to take care of my parents, I cannot quit and let others take care of my parents when they need care
	If I have no choice, I'll quit my job and care for my parents	

Both the Chinese and Japanese participants believed that children should take responsibility for parental care. The Japanese participants believed that the eldest child should be more responsible and stressed that although gender equality is widely recognized in Japanese society, the impression remained that females should do the care. They also felt that it was socially recognized that more women than men should quit their jobs to care for their parents and that women must take responsibility. However, all Chinese participants believed that regardless of gender or birth order, all children should be equally responsible for parental care.

I think it is the responsibility of every child to fulfill his or her filial duty... I think children have a responsibility and duty to look after the parents who raised them, but this should not be the sole responsibility of one person. (C-3)
When I became an older brother, I was told that I had to do things well, or something like that....After all, I am the older brother, so I think on my own involuntarily that I have to do everything right, that I have to support the family, and that I have to take care of my parents.... (J-2)
Even in terms of work, when it comes to the choice of quitting a job and caring for the family, I cannot really imagine the man quitting, but I think someone would say that I (a woman) should quit my job and care for the family. (J-10)

The participants from both China and Japan stated that they wanted to care for their parents to repay them for raising them.

My parents raised me and put in so much effort and went through so much suffering, so I think as a child I need to reward them. (C-8)
I owe my parents a favor for raising me, and I don't think forcing them into a nursing home would be a good filial duty. Of course, I think parents want their children to take care of them as a way of repaying the favor they have done for us, too. (J-17)

The participants from both China and Japan stated that they were influenced by their surroundings to care for their parents. The Chinese participants explained that they were stressed by traditional norms and the Japanese participants emphasized the influence on their reputation and others' expectations of their role as a child.

From the perspective of traditional norms, it is unfilial to leave my parents in a nursing home, so if I leave my parents to others, my parents would be very resistant, so I want to take care of them by myself. (C-18)
If I decide to leave my parents at a nursing home,...rumors or some bad impressions (like the impressions imposed by society) would be spread by the people around me, which would make me feel uncomfortable. (J-19)

The Chinese participants stated that family was more important than work. If they could not balance parental care and their work, they would quit their jobs. The Japanese participants stated that if they were unable to balance caring for their parents with their work, they would keep their jobs and leave care to others.

I am related to my parents by blood, so if there is a conflict (between work and caring for my parents), I think I would have to compromise and devote myself to caring for my parents. (C-16)
I think my parents' opinion is important, I will try my best to take care of them, but when I cannot afford it or they need to be taken care of by others because of some of my personal issues, I would try my best to persuade them to move to a nursing home. (J-10)

In addition, this metatheme showed the responsibility of parental care for both Chinese and Japanese participants due to the repayment of parents' upbringing and influences from their surroundings. However, when comparing jobs and parental care, Chinese participants tended to take care of their parents, while Japanese would like to keep their job. On the other hand, Chinese participants emphasized equal responsibility for all children, and Japanese participants tended to believe women and first-born children should take up the responsibility.

#### 3.2.3. Importance of parent-child interactions about parental care

This metatheme comprised two sub-metathemes (Table 4).

**Table 4:** Metatheme 3: importance of parent-child interactions about parental care.

**Sub-metathemes**	**Themes**	
	**China**	**Japan**
Consideration for parents' feelings about their own care	I want to take care of my parents by myself because I think my parents are not comfortable leaving them to others.	I don't want my life to be determined like this anymore, so although it is painful, at least I will get a home helper.
	I want to care for my parents myself because they may feel abandoned if I leave them to others	I'll follow my parents' advice, but if I care for them all the time, I'll tell them to move into a nursing home
Discussions with parents about their own care	If there is a conflict with parents, I will respect and compromise with their opinions	My parent's opinion is important to me, but I want to continue working and I want them to be in a nursing home when they need care
	It is important to respect parents' opinions and be as satisfied as possible	In fact, when I'm in that situation, I'd like to interact with my parents and try to convince them

Both Chinese and Japanese participants stated that their parents' feelings were important. The Chinese participants stated that if they left the parental care to others, both their parents and themselves would feel resistance and loneliness, so they preferred to care for their parents by themselves as much as possible. The Japanese participants stated that although the parents' feelings were important, they also had their own lives and careers; therefore, even though it would be heartbreaking, they'd leave their parents to others.

I would like to care for my parents myself as much as possible because if I care for my parents myself, it would provide psychological comfort, and if they were cared for by a caregiver, I believe my parents would feel alone. (C-2)
No matter how much the parents hope for their children's care, absolutely their children still have their own lives. Although it is a little bit painful, at least, I think I would get a home helper or other services. (J-14)

Both the Chinese and Japanese participants emphasized the importance of seeking their parents' opinions about their care. If there was a conflict with the parents, the Chinese participants stated that they would compromise and the Japanese participants stated that they would seek to convince their parents.

I would respect my parent's opinions if my thoughts on care diverged from theirs. Anyway, parents' feelings are the priority in care, and I want to satisfy their wishes as much as possible as a child. If we cannot work it out, I think I would do what they want. (C-13)
I want to respect my parents' feelings and opinions, of course, but I don't want to force myself to cater to them, so I'd persuade them until they agreed with my opinions. (J-8)

In addition, this metatheme highlighted the importance of interactions between generations. Both Chinese and Japanese participants emphasized communication with parents, however, Chinese participants were more parent-centered, which means they would follow them as far as possible; whereas Japanese participants were more self-centered to convince their parents to agree with their ideas.

**Figure 1:**
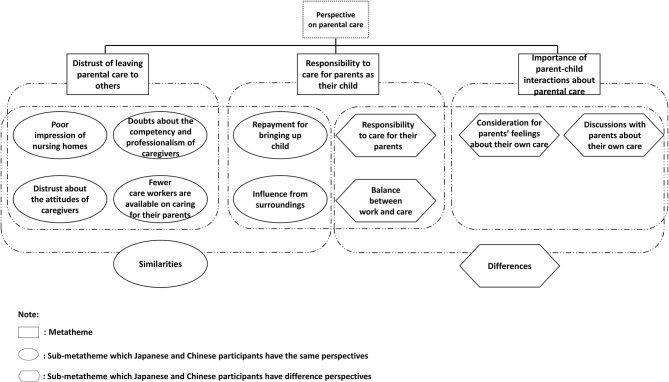
Perspectives on parental care between Chinese and Japanese university students.

In summary, there were both similarities and differences in perspectives on parental care between Chinese and Japanese participants (Figure 1). Both Chinese and Japanese participants shared a similar perspective of distrust toward leaving parental care to others and taking care of their parents for repayment for upbringing and surroundings' influence. However, there were also some differences between Chinese and Japanese participants. Japanese participants tended to insist on their own ideas and convince their parents in continue their work and communicate with their parents, while Chinese participants tended to follow their parents' opinions. Furthermore, Japanese participants emphasized the importance of the first-born child and believed that women should take on more responsibility for parental care due to cultural background. In contrast, Chinese participants considered gender and birth order inequalities to be outdated and emphasized equality for all children.

## 4. Discussion

To provide some guidance on the future needs of the healthcare society, Chinese and Japanese university students were asked to give their perspectives on parental care in focus groups, from which three metathemes were identified: distrust of leaving parental care to others, responsibility to care for parents, and importance of parent-child interactions about parental care.

### 4.1. Similarities and differences on perspective of parental care

Both the Chinese and Japanese student groups had a mistrust of nursing homes because of news about the mistreatment of older adults. A Japanese Ministry of Health, Labor and Welfare (MHLW) publication, the “Act on the prevention of abuse on older adults and support for caregivers of older adults,” reported that in 2020, 2,097 abuse cases on older adults had been referred and 595 judgments had been made (32). Because of the public distrust in nursing homes, a system is being developed in Japan to prevent abuse on older adults in nursing care facilities, which is hoped will ease resistance to the system and improve how the public views nursing homes (33). In China, attitudes toward nursing homes have shifted from it being a stigma to recognizing that nursing homes can provide high-cost professional care; nonetheless, some negative attitudes remain (34). It has also been reported that Chinese nursing homes are of low quality and have a low capacity to accommodate the older adults in China (35). For these reasons, putting parents in a nursing home is seen as unfilial, and many parents resist moving there.

The results from this study indicated that increasing the number and professionalism of nursing home caregivers were required, which agreed with a previous study that found nursing home caregivers in China are often poorly educated older adults who lack expertise (36). Although great efforts have been made to improve care services in nursing homes and require that healthcare workers have formal occupational training, there is a shortage of healthcare workers in China (37). As a result, the lack of care skills and the poor quality of medical care have received widespread attention (38). Even though the Chinese government has published “basic specifications of service quality in senior care organization,” careworker in nursing homes was found underqualified for care work than other Western countries (35). Careworker job vacancies by prefecture in Japan are on average 3.97 times higher than the number of care workers employed in nursing homes (39). The MHLW estimated that 2.43 million care workers would be needed by 2025; however, the expected number is estimated at only 2.11 million, a potential shortage of 320,000 (40), which could lead to increased careworker workloads, which in turn could reduce the quality and safety of care.

In East Asian cultures, the firstborn is expected to live with their parents after marriage, and the firstborn and their partner are expected to take care of their parents (41). This study found that this was still the belief of most Japanese university students; however, the Chinese students felt that the responsibilities lay with all children regardless of their birth order. As Chinese caregivers are suffering from emotional and financial stress, sharing the care responsibilities would be easier (42). Nonetheless, although female work opportunities have increased in Japan and the expectations of care by the family have weakened, females were seen as being more likely to be the primary caregivers (43). However, the responsibilities are more equal in China because of its socialist system and legislation specifying (44). Further, because females have gained greater access to higher education in China, they are less likely to abandon their careers to take care of their aging parents and more likely to insist upon shared sibling responsibility (43).

Filial piety emphasizes dependence, obligation, and reciprocity in intergenerational relationships, all of which should strengthen intergenerational bonds (45). This study found that the Chinese participants were more likely to leave their jobs if they were unable to balance work and parental care, whereas the Japanese participants were more likely to place their parents in a nursing home. China has strengthened the social obligations of children to care for their aging parents (46); and in many Chinese families, older people continue to have strong bonds with their children and are involved in their daily lives, such as taking care of grandchildren and providing financial support, while they can also receive necessary care from their children as an exchange (47). Since the promulgation of Japan's long-term care insurance system, the family role has shifted from the direct provision of care to an organizational and administrative role and emotional support when using the available resources, which had led to a lower tendency for children to quit their jobs to take up parental care (48).

The Japanese and Chinese university student groups both stated that parent-child interactions were essential to strengthen parent-child connections and parental health. In China, Confucian filial piety was the guiding principle, which required children to ensure the emotional and physical wellbeing of their aging parents (49). Confucian culture means that older Chinese and their children have close emotional ties and high mutual emotional dependence (50). If their parents were resistant to entering a nursing home, both groups claimed that they would respect their parent's wishes (51). In Japan, however, because of the healthcare insurance scheme and economic growth, older parents tend to be more independent and less in need of their children's support; therefore, the participants said that they would seek to persuade their parents to enter a nursing home rather than compromise (52).

### 4.2. Practical implications

Based on the study's findings, several practical suggestions are given for improving healthcare systems in China and Japan. First, studies in Japan and the US found that the quality of long-caregiver services at nursing homes was poor (32, 53). Therefore, care service delivery and healthcare systems must be improved to build trust between healthcare professionals and the public. Second, because of cultural contexts such as filial piety, aging policies can have profound implications for both older adults and their children. Therefore, services and interventions must be developed to ensure the wellbeing and livelihoods of both the children and the parents. Third, caregiver gender equality, especially in Japan needs to be promoted (54). Fourth, similar to Japan, China needs formal policies such as nursing care leave in place to provide a better work and life balance for Chinese caregivers, and supports to mitigate the dissonance between their parental caregiver roles and their own lives.

### 4.3. Limitations

This study had some limitations. First, because only university students were included in the study, it was difficult to extrapolate the results to all people in the younger generations. Further, as none of the participants had yet experienced the difficulties of balancing parental care and work obligations, the findings were related to the potential worries they may have in the future. Therefore, more studies are required to determine the reactions to these problems in the coming decades. While it was found that the university students were willing to help with parental care, more quantitative research is needed because of the small sample size and the qualitative research frame. Future research should explore the perspectives of all younger generations on parental care. It could also be useful to examine the attitudes of other generations to explore generational differences. On the other hand, exploring the socio-cultural reasons influencing parental care perspectives, or comparing the differences in institutional systems for older adults across different nations is important. Such research can assist policymakers in alleviating potential concerns.

### 4.4. Conclusions

This study examined the attitudes of Chinese and Japanese university students toward parental care. The findings suggested that both countries needed to improve their care service delivery and healthcare systems and build trust between healthcare professionals and the public. Support will also be needed to assist children to balance their care responsibilities with their work and family life. Culturally, it was found that healthcare policies had significant social implications for older adults and their children; therefore, future service delivery policies need to consider the wellbeing of both the children and the parents.

## Data availability statement

The raw data supporting the conclusions of this article will be made available by the authors, without undue reservation.

## Ethics statement

The studies involving humans were approved by the Ethics Committee of Epidemiology Research at Hiroshima University (E-2633-2). The studies were conducted in accordance with the local legislation and institutional requirements. The participants provided their written informed consent to participate in this study. Written informed consent was obtained from the individual(s) for the publication of any potentially identifiable images or data included in this article.

## Author contributions

XP played a central role in the research, planning the research, collecting data, analyzing and interpreting it, and writing the manuscript. HN carried out research planning, analysis, and interpretation and contributed significantly to the writing of the manuscript. YI interviewed the participants and analyzed and reviewed the manuscript. HC contributed to the analysis, interpretation, and writing of the manuscript and critically reviewed the manuscript. FS contributed to the analysis and interpretation and writing of the manuscript. MY contributed to the writing of the manuscript and critically reviewed the manuscript. RL contributed to the writing and the editing of the manuscript. All authors read and approved the final manuscript.
